# Population Snapshot of *Streptococcus pneumoniae* Causing Invasive Disease in South Africa Prior to Introduction of Pneumococcal Conjugate Vaccines

**DOI:** 10.1371/journal.pone.0107666

**Published:** 2014-09-18

**Authors:** Kedibone M. Ndlangisa, Mignon du Plessis, Nicole Wolter, Linda de Gouveia, Keith P. Klugman, Anne von Gottberg, for GERMS-SA

**Affiliations:** 1 Centre for Respiratory Diseases and Meningitis (CRDM), National Institute for Communicable Diseases (NICD), a division of the National Health Laboratory Service, Johannesburg, South Africa; 2 Respiratory and Meningeal Pathogens Research Unit, University of the Witwatersrand, Johannesburg, South Africa; 3 Medical Research Council, Johannesburg, South Africa; 4 Hubert Department of Global Health, Rollins School of Public Health, and Division of Infectious Diseases, School of Medicine, Emory University, Atlanta, Georgia, United States of America; Fondazione IRCCS Ca’ Granda Ospedale Maggiore Policlinico, Università degli Studi di Milano, Italy

## Abstract

We determined the sequence types of isolates that caused invasive pneumococcal disease (IPD) prior to routine use of pneumococcal conjugate vaccines (PCV) in South Africa. PCV-13 serotypes and 6C isolates collected in 2007 (1 461/2 437, 60%) from patients of all ages as part of on-going, national, laboratory-based surveillance for IPD, were selected for genetic characterization. In addition, all 134 non-PCV isolates from children <2 years were selected for characterization. Sequence type diversity by serotype and age category (children <5 years vs. individuals ≥5 years) was assessed for PCV serotypes using Simpson’s index of diversity. Similar genotypes circulated among isolates from children and adults and the majority of serotypes were heterogeneous. While globally disseminated clones were common among some serotypes (e.g., serotype 1 [clonal complex (CC) 217, 98% of all serotype 1] and 14 [CC230, 43%)]), some were represented mainly by clonal complexes rarely reported elsewhere (e.g., serotype 3 [CC458, 60%] and 19A [CC2062, 83%]). In children <2 years, serotype 15B and 8 were the most common serotypes among non-PCV isolates (16% [22/134] and 15% [20/134] isolates, respectively). Sequence type 7052 and 53 were most common among serotypes 15B and 8 isolates and accounted for 58% (7/12) and 64% (9/14) of the isolates, respectively. Serotype 19F, 14, 19A and 15B had the highest proportions of penicillin non-susceptible isolates. Genotypes rarely reported in other parts of the world but common among some of our serotypes highlight the importance of our data as these genotypes may emerge post PCV introduction.

## Introduction

In South Africa, a country with a high prevalence of HIV infection, *Streptococcus pneumoniae* is an important cause of disease [1]. In 1997–1998, the rate of invasive pneumococcal disease (IPD) among HIV-infected South African children <2 years of age, was estimated to be 42 times higher than among HIV-uninfected children [2]. Moreover, adults infected with HIV are more likely to be infected with pediatric serotypes and antibiotic-resistant pneumococci [1], [3].

The first generation 7-valent pneumococcal polysaccharide-protein conjugate vaccine (PCV-7) has significantly reduced vaccine-serotype IPD among vaccinated children in countries where it has been introduced [4]–[6]. Reduction of disease in unvaccinated children and adults because of herd effect was also reported in those countries [5]. Despite the benefits of PCV-7, an increase in disease due to non-vaccine serotypes, mainly serotype 19A, has been reported in some countries where the vaccine is used routinely [4]–[8]. Other serotypes that increased post PCV-7 introduction include 1, 3, 7F and 22F [4], [5]. While these increases were mainly due to the expansion of pre-existing clones, the emergence of sequence types previously associated with PCV-7 serotypes have also been reported among non-PCV-7 serotypes [9]. In the United States, clonal complex (CC) 320/271, which was associated with multidrug resistant serotype 19F isolates prior to PCV-7 introduction, emerged among serotype 19A isolates following PCV-7 introduction [9].

Natural fluctuations in circulating genotypes in the absence of any known interventions have also been shown to occur [10] and factors other than vaccine pressure can result in proliferation of pneumococcal strains. This was demonstrated by an increase in antibiotic-resistant serotype 19A in Spain, Korea and Israel before routine use of PCV-7 [11]–[13]. Baseline data describing the genetic structure of pneumococci prior to the implementation of new interventions such as conjugate vaccines are therefore required to detect genotype changes potentially resulting from such interventions.

In South Africa, PCV-7 was registered in 2005 but was initially only available in the private healthcare sector. The vaccine was introduced into the childhood immunization programme in April 2009 as a novel 2+1 schedule administered at 6, 14 and 40 weeks of age [14] and was replaced by PCV-13 in June 2011. Limited molecular characterization data are available for pneumococcal isolates from South Africa. We therefore used multilocus sequence typing (MLST) to investigate the clonal composition of invasive pneumococci prior to routine use of pneumococcal conjugate vaccines.

## Materials and Methods

### National surveillance

Isolates were collected as part of the GERMS-SA (Group for Enteric, Respiratory and Meningeal Disease Surveillance) national, laboratory-based surveillance for IPD in South Africa, initiated in 1999 [15]. Isolates and patient data were submitted to the National Institute for Communicable Diseases from 187 participating laboratories across all nine provinces. A case of IPD was defined as the isolation of *S. pneumoniae* from a normally sterile site specimen (e.g., blood, cerebrospinal fluid [CSF], pleural fluid, joint fluid). Isolates were received on Dorset transport media (Diagnostic Media Products [DMP], Johannesburg, South Africa) and sub-cultured on arrival at the reference laboratory, on 5% horse blood agar plates (DMP) in the presence of an optochin disc (Mast Diagnostics, Virginia, USA). Pure cultures were stored in 10% skim milk (DMP) at −70°C. In addition, cases included patients with normally sterile site specimens testing positive by PCR, or bacterial latex antigen supported by Gram stain microscopy [16]. Confirmed unreported cases identified through audits of databases in participating laboratories were also included. In 2003, national surveillance was enhanced to include additional data such as patient outcome and HIV serological status from approximately 30 sentinel sites in all nine provinces. Surveillance reporting increased from 2003 onwards, and stabilized by 2005 [17]. Isolates for this study were selected from cases reported in 2007, a stable, pre-vaccine year.

### Serotyping and antimicrobial susceptibility testing

Serotypes were determined by the Quellung method using type-specific antisera (Statens Serum Institut, Copenhagen, Denmark) [18]. Isolates were initially screened for susceptibility to oxacillin (for penicillin), tetracycline, chloramphenicol, erythromycin, clindamycin, rifampicin and trimethoprim-sulfamethoxazole by disc diffusion (Mast Diagnostics Group Ltd., Merseyside, UK). Isolates testing non-susceptible on disc screening had minimum inhibitory concentrations (MIC) determined using agar dilution or E-test (AB BIODISK, Solna, Sweden). Interpretation of results was done according to Clinical and Laboratory Standards Institute guidelines and breakpoints [19]. Isolates were considered non-susceptible to penicillin at MICs ≥0.12 mg/L using the oral penicillin meningitis breakpoints. For other antimicrobials, isolates were defined as non-susceptible if they were intermediately or fully resistant to the agent tested, and isolates were classified as multidrug resistant if they were non-susceptible to penicillin plus two or more other classes of antimicrobial agents tested.

### Strain selection for MLST

Isolates were selected from cases reported in 2007. PCV serotypes (1, 3, 4, 5, 6A, 6B, 7F, 9 V, 14, 18C, 19A, 19F and 23F) and 6C were grouped by patient age category (children <5 years [n = 927] and older individuals ≥5 years [n = 1 510]). Sampling was conducted as follows: for serotypes represented by ≤100 isolates per serotype, per age category, all isolates were included for characterization. Due to the large numbers of isolates available for certain serotypes (4, 6A, 6B, 14, 19F, 23F) sampling was rationalized by selecting 50% where numbers exceeded 100 (per serotype and per age category) (Table 1). Isolates were sorted by collection date and every second isolate was selected. In addition, all non-PCV serotype isolates causing disease in 2007 among children <2 years of age were analyzed (n = 134).

**Table 1:** Clonal complex and sequence type distribution of pneumococcal conjugate vaccine serotype isolates causing invasive pneumococcal disease in South Africa, 2007 (n = 1 064).

Serotype	Clonal complex	Sequence type
(No. ofisolates)	(No. of isolates)	(No. of isolates)
1 (146)	217 (143)	217 (111), 612 (31), 3577 (1)
	2296 (3)	611 (3)
3 (93)	458 (56)	458 (44), 6366 (1), 7348 (1), 7349 (1),7350 (6), 7353 (3)
	180 (14)	180 (6), 3842 (1), 4118 (2), 7356 (1),7357 (4)
	378 (11)	378 (1), 7362 (1), 7363 (1), 7364 (1), 7365(1), 7367 (1), 7368 (1), 7369 (3), 7366 (1)
	Other (12)	700 (1), 1765 (1), 2837 (5), 7347 (1), 7351 (1),7352 (1), 7378 (1), 7997 (1)
4 (81)	32 (31)	5410 (16), 5655 (4), 6367 (1), 6368 (1), 7370 (4), 7372 (1), 7373 (1), 7374 (1), 7375 (1),
		7376 (1)
	Other (50)	63 (1), 205 (3), 271 (1), 1221 (25), 1222 (6),2213 (1), 2290 (5), 6369 (1), 7354 (1), 7355
		(2), 7358 (1), 7359 (1), 7371 (1), 7727 (1)
5 (18)	289 (18)	289 (3), 5659 (12), 6370 (1), 7377 (1), 7745 (1)
6A (95)	473 (37)	1096 (1), 2285 (10), 2295 (2), 4087 (3), 5073 (4), 6372 (2), 6373 (1), 6374 (1), 6380 (1), (1),
		6381 (1), 6382 (1), 6385 (1), 6388 (1), 6390 (1), 7298 (1), 7300 (1), 7301 (1), 7310 (1), 7735
		7736 (1), 7744 (1)
	156 (23)	138 (2), 185 (5), 1447 (1), 2987 (1), 3207 (1),6375 (1), 6376 (1), 6379 (2), 6389 (3), 7304
		(1), 7307 (1), 7309 (1), 7313 (1), 7740 (1),7741 (1)
	1094 (19)	1094 (5), 2289 (7), 4941 (2), 6371 (1), 6383 (1), 6384 (2), 6387 (1)
	Other (16)	2283 (1), 2909 (1), 5251 (1), 5412 (1), 6377 (1), 6378 (1), 6386 (1), 6391 (1), 7084 (1), 7299
		(1), 7306 (1), 7308 (1), 7312 (1), 7721 (1),7732 (1), 7743 (1)
6B (113)	2421 (26)	2421 (12), 2909 (2), 4929 (2), 6290 (1), 6291 (1), 6299 (1), 6307 (1), 6309 (2), 7305
		(1), 7724 (1), 7731 (1), 8231 (1)
	156 (26)	138 (1), 185 (7), 1447 (1), 3473 (1), 6235 (1),6259 (1), 6293 (3), 6296 (1), 6297 (5), 6304
		(1), 6526 (1), 6529 (1), 7302 (1), 7738 (1)
	473 (12)	2285 (2), 4087 (4), 5073 (1), 6308 (2), 6289 (1), 7303 (1), 7311 (1)
	1094 (8)	1094 (4), 2289 (1), 6292 (1), 4941 (1), 6298 (1)
	242 (6)	242 (2), 6279 (1), 6288 (1), 6303 (1), 6305 (1)
	1381 (4)	6239 (2), 6243 (2)
	230 (4)	230 (2), 6231 (1), 6234 (1)
	Other (27)	193 (1), 439 (1), 2185 (1), 3594 (1), 6230 (1),6232 (1), 6233 (1), 6236 (2), 6245 (1), 6251
		(2), 6276 (1), 6278 (1), 6294 (2), 6295 (1), 6300 (1), 6301 (1), 6302 (1), 6306 (2), 6310 (1),
		6393 (1), 6525 (1), 7084 (1), 7733 (1)
6C (11)	2185 (9)	2185 (3), 2283 (1), 6310 (3), 6311 (1), 7345 (1)
	Other (2)	6297 (1), 7344 (1)
7F (29)	218 (21)	218 (8), 3544 (9), 6838 (2), 7884 (1), 7886 (1)
	Other (8)	2416 (1), 7048 (1), 7360 (1), 7361 (1), 7379 (1), 7883 (1), 7885 (1), 7887 (1)
9V (70)	4881 (40)	3454 (14), 4881 (22), 6314 (1), 6316 (1),6530 (1), 7889 (1)
	156 (19)	156 (6), 162 (4), 557 (1), 609 (5), 644 (1),2280 (1), 7891 (1)
	Other (11)	53 (1), 473 (1), 706 (1), 3983 (1), 5408 (1),6312 (1), 6313 (1), 6315 (1), 6531 (1),7888 (1),
		7890 (1)
14 (115)	230 (49)	230 (25), 1701 (1), 2707 (4), 3980 (1),6227 (1), 6228 (1), 6229 (1), 6231 (2), 6234 (7), 6532
		(1), 7267 (2), 7283 (1), 7291 (2)
	15 (30)	1492 (11), 2652 (7), 7282 (2), 7271 (1),7276 (1), 7278 (1), 7280 (1), 7284 (1), 7287 (1)
		7288 (1), 7726 (1), 7728 (1), 7729 (1)
	63 (25)	63 (7), 861 (2), 2414 (6), 5004 (1), 5187 (1), 7270 (1), 7277 (1), 7279 (1), 7281 (1), 7293
		(1), 7722 (1), 4902 (2)
	Other (11)	124 (1), 2421 (1), 6226 (1), 6393 (1), 6533 (1),7266 (1), 7269 (1), 7273 (1), 7292 (1), 7295
		(1), 8233 (1)
18C (57)	1016 (17)	102 (4), 1016 (3), 6245 (2), 6247 (1), 7262 (2), 7265 (2), 7290 (1), 7297 (1), 7730 (1)
	1381 (13)	6239 (5), 7272 (2), 7274 (1), 7275 (3), 7289 (1), 6240 (1)
	193 (12)	4893 (1), 6241 (1), 6242 (4), 6246 (1), 6248 (1), 7268 (1), 7723 (1), 6237 (1), 7263 (1)
	6236 (10)	6236 (6), 7264 (1), 7286 (1), 7296 (1), 7742 (1)
	Other (5)	6238 (1), 6244 (1), 6250 (1), 7294 (1), 7346 (1)
19A (131)	2062 (109)	2062 (76), 4872 (1), 6121 (5), 6253 (1), 6254 (1), 6255 (3), 6256 (2), 6258 (1), 6260 (1),
		6261 (1), 6262 (2), 6263 (1), 6264 (1), 6266 (1), 6268 (1), 7716 (1), 7718 (1), 7720 (1), 7894
		(1), 7895 (1), 7897 (2), 7898 (1), 7899 (1),7903 (1), 8230 (1)
	156 (9)	75 (1), 172 (4), 6265 (1), 7734 (1), 7737 (1),7861 (1)
	Other (13)	1381 (1), 2416 (1), 2421 (1), 6257 (1), 6267 (1), 7715 (1), 7717 (1), 7739 (1), 7896 (1), 7900
		(1), 7901 (1), 7902 (1), 7904 (1)
19F (46)	320 (23)	271 (16), 1467 (1), 6270 (4), 6275 (1),7725 (1)
	347 (6)	347 (6)
	420 (5)	420 (2), 3217 (1), 7857 (1), 7858 (1)
	Other (12)	81 (1), 230 (1), 2067 (2), 6271 (1), 6272 (1),6273 (1), 6278 (1), 6281 (1), 7131 (1), 7719
		(1), 7860 (1)
23F (59)	242 (25)	2059 (5), 5653 (5), 6279 (5), 6285 (2), 242 (1),362 (1), 6280 (1), 6284 (1), 7862 (1), 7868
		(1), 8232 (1), 8233 (1)
	156 (12)	172 (5), 361 (1), 1535 (1), 6281 (1), 6287 (2),7861 (1), 7882 (1)
	Other (22)	81 (4), 193 (1), 230 (1), 320 (1), 439 (2), 802 (2), 4087 (1), 63 (1), 6286 (1), 6389 (1), 7863
		(1), 7865 (1), 7866 (1), 8032 (1), 8033 (1),8034 (1), 8035 (1)

### MLST

DNA was extracted directly from 100 µl of stored culture in 10% skim milk using a MagNA Pure LC 2.0 instrument with the MagNA Pure LC DNA Isolation kit III (Roche, Mannheim, Germany) according to the manufacturer’s instructions. Extracted DNA was eluted into 100 µl of elution buffer and stored at −20°C. If poor quality sequencing results were obtained, DNA was re-extracted by boiling pure overnight cultures suspended in 100 µl of sterile deionized water for 10 minutes at 95°C. MLST was performed as previously described [20], using primer pairs described by the Centers for Diseases Control and Prevention, Atlanta, USA (http://www.cdc.gov/ncidod/biotech/strep/alt-MLST-primers.htm) or primers listed on the MLST website (http://spneumoniae.mlst.net/). MLST genes were sequenced in one direction using the Big Dye Terminator v3.1 cycle sequencing kit (Applied Biosystems [ABI], Foster City, CA, USA), and products were analyzed using the ABI 3500 genetic analyzer. Sequences were aligned and analyzed using Lasergene software v9 (DNASTAR, Wisconsin, USA) and compared with sequences on the global MLST database (http://spneumoniae.mlst.net/) to assign allele numbers and ST. For new alleles, amplicons were sequenced in both directions and trace files were submitted to the MLST database curator. New profiles arising as a result of new alleles were also submitted to the curator for assignment of a sequence type.

### Data analysis

The eBURST v3 algorithm available on the MLST website was used to determine relationships between sequence types (ST) and to generate population snapshots [21]. The minimum number of identical loci was set at 0 for group definition. Single- (SLV) and double-locus (DLV) variants were defined as ST’s differing from each other by one and two alleles, respectively. The default bootstrap (1 000) was used to assign the group founder ST. A clonal complex was defined as a group of ST’s sharing at least 6 of 7 identical alleles with another ST in the group. Clonal complexes were named after the group founder ST as predicted by eBURST when using all data available in the MLST database. Singletons were defined as unrelated sequence types that did not belong to any clonal complex. Isolates of the same clonal complex or sequence type but with different serotypes were presumed to be capsular switch variants.

Sequence type diversity by serotype and age category (children <5 years of age and individuals ≥5 years) was assessed using Simpson’s index of diversity (D). The value of D ranges from 0 to 1, and the greater the value the greater the sample diversity. Analysis was performed through an online tool for diversity and partition congruency calculations (http://darwin.phyloviz.net/ComparingPartitions/index.php?link=Tool). For this assessment, only PCV serotypes were included.

### Ethics

The surveillance study and molecular characterization of isolates were approved by the Human Research Ethics Committee, University of the Witwatersrand, South Africa (protocol numbers: M081117 and M111008).

## Results

### National IPD surveillance

A total of 4 733 cases of IPD were reported from January through December 2007, of which, 70% (3 329/4 733) had viable isolates available for further characterization. The majority of cases with viable isolates were diagnosed from positive blood cultures (1 879/3 329, 56%), followed by CSF (1 005/3 329, 30%). In addition, isolates were recovered from both blood and CSF (247/3 329, 7%), pleural fluid (1 44/3 329, 4%) or other normally sterile site specimens (54/3 329, 2%).

**Figure 1:**
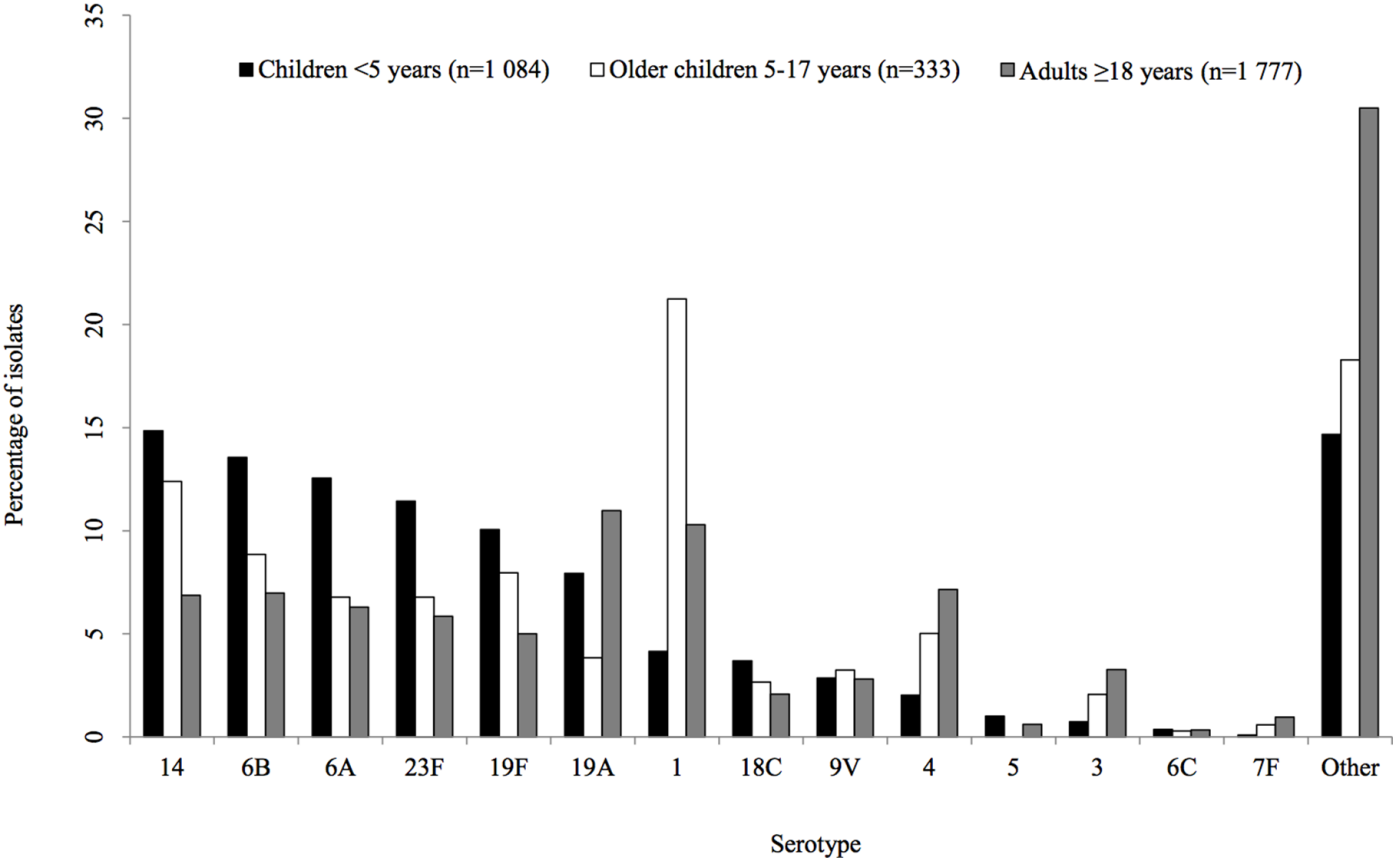
Distribution of pneumococcal serotypes causing invasive disease in South Africa, by age group, 2007 (n = 3 194). ‘Other’ indicates serotypes not included in pneumococcal conjugate vaccines (PCV-13 and 6C).

Among cases with known gender (97% [3 241/3 329]), females and males were equally represented (1 638/3241, 51% and 1 603/3241, 49%, respectively). Age was known for 96% (3 194/3 329) of cases with viable isolates, and of these, 34% (1 084/3 194) were children <5 years, 10% (333/3 194) were older children 5–17 years and 56% (1 777/3 194) were adults ≥18 years. Overall, PCV serotypes were responsible for 86% (927/1 084), 82% (273/333) and 69% (1 235/1 777) of IPD in children <5 years, older children 5–17 years and adults ≥18 years, respectively. Serotypes 14, 6B and 6A were the most prevalent serotypes among children <5 years accounting for 41% (446/1 084), while 1, 14 and 6B were the most common serotypes among older children 5–17 years (135/333, 41%) and 1, 19A and 4 were prevalent among individuals ≥18 years (505/1 777, 28%) (Figure 1). Among children <2 years, serotype 15B and 8 were the most common serotypes among non-PCV serotypes, and represented 16% (22/134) and 15% (20/134) of the isolates, respectively. The remaining isolates (69%, 92/134) belonged to 32 serotypes. Non-susceptibility to penicillin was detected in 43% (1429/3 329), of which 39% (562/1 429) were multidrug resistant. HIV status was known for 32% (1 064/3 329) of cases with viable isolates, the majority of whom were HIV infected (816/1 064, 77%). Overall, PCV serotypes were responsible for 77% (633/816) and 81% (186/231) of IPD in HIV infected and uninfected cases, respectively (p = 0.002).

### Multilocus sequence typing (MLST)

A total of 1 461 isolates representing PCV serotypes and 6C (1 461/2 437, 60%) were selected for MLST (Table S1), of which complete results were available for 73% (1 064/1 461). Poor sequence data or PCR amplification failure for some of the genes resulted in incomplete MLST results for some isolates, 43% (169/397) of which were from children <5 years. MLST results were available for 33% (269/816) of isolates from HIV infected and 37% (85/231) of isolates from HIV uninfected individuals. All 134 isolates representing non-PCV serotypes, from children <2 years of age were selected for characterization and complete results were obtained for 55% (74/134) of these.

### MLST genotypes

**Figure 2:**
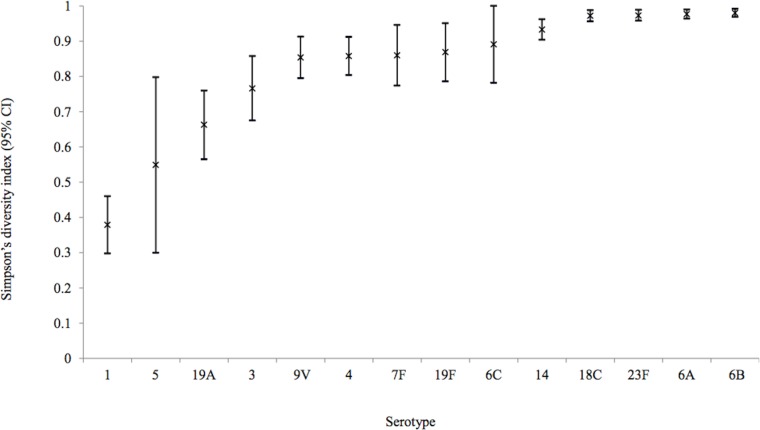
Diversity of sequence types within serotypes. Simpson’s index of diversity was used to assess diversity of isolates that caused invasive pneumococcal disease in 2007 among individuals of all ages, South Africa.

Data are summarized in Table 1 and eBURST population snapshots, by serotype, are presented as supporting information (Figures S1 to S14). For PCV serotypes, similar clonal complexes were found among isolates from children and older individuals and there were no differences in sequence type diversities between the two age groups (data not shown). Simpson’s diversity indices were therefore calculated for each serotype using combined sequence type data from isolates of children and adults (Figure 2). The majority of serotypes were heterogeneous, however, serotypes 1 and 5 were the least diverse (D = 0.38 and 0.55, respectively) while 6A, 6B, 18C and 23F were the most diverse serotypes, with D ranging from 0.97 for 18C and 23F to 0.98 for 6A and 6B. The majority of serotype 1 isolates were CC217 (143/146, 98%). CC458, which was the largest clonal complex among serotype 3 isolates, represented 60% (56/93) of the serotype 3 isolates. The main clonal complex among serotype 4 isolates was CC32 (31/81, 38%). All serotype 5 isolates were CC289. For serotype 6A, 3 common clonal complexes were identified, of which the most prevalent, CC473, represented 39% (37/95) of the isolates. Several complexes were identified among serotype 6B isolates, of which CC2421 (26/113, 23%) and CC156 (26/113, 23%) were the most common. CC2185 represented majority of serotype 6C isolates (9/11, 82%). CC218 represented 72% (21/29) of serotype 7F isolates. The major clonal complex (CC4881) among serotype 9 V isolates represented 57% (40/70) of the isolates. Of the clonal complexes identified among serotype 14 isolates, CC230 was the largest and represented 43% (49/115) of the isolates. Serotype 18C isolates were differentiated into 4 main clonal complexes. The largest clonal complex among serotype 18C isolates (CC1016) represented 30% (17/57) of the isolates. The majority of serotype 19A isolates were CC2062 (109/131, 83%). CC320 was the most prevalent clonal complex among serotype 19F isolates (23/46, 50%). For serotype 23F, CC242 (25/59, 42%) and CC156 (12/59, 20%) were the major clonal complexes. For non-PCV serotypes, ST7052 which belong to CC346, was predominant (7/12, 58%) among serotype 15B isolates, while ST53 (CC62) was prevalent (9/14, 64%) among serotype 8 isolates (Table 2).

**Table 2:** Sequence type distribution among pneumocoocal non-conjugate vaccine serotype pneumococci causing invasive disease in South African children <2 years (n = 74), 2007.

Serotype	Sequence type(no. of isolates)	Sequence type (other serotypeslisted in the MLSTdatabase)^1^
15B (12)	411 (1), 4693 (1), **5808** (1),**7052 (7),** 8687 (1), 8820 (1)	4693 (19A), 8687 (6A)
8 (14)	53 (9), 1480 (1), 3847 (2), 7888 (1), 8831 (1)	7888 (9V)
9N (6)	66 (2), 3983 (2), 5624 (2)	66 (4, 14. 19A, 19F, 23F)
16 (4)	4088 (2), 8827 (1), 8834 (1)	none
10A (4)	2068 (1), 8821 (1), 8825 (1),8839 (1)	none
29 (4)	5483 (1), 8826 (3)	none
13 (3)	5647 (3)	none
12F (3)	989 (1), 2416 (1), 8828 (1)	2416 (7F, 19A)
34 (2)	8818 (1), 8822 (1)	none
17F (2)	8829 (1), 8835 (1)	none
7C (2)	8838 (2)	none
23A (2)	2319 (1), 5080 (1)	5080 (23F)
33D (1)	8714 (1)	8714 (6A)
33F (1)	8837 (1)	none
35B (1)	8830 (1)	none
9L (1)	8833 (1)	none
NT (1)	**344 (1)**	344 (6A, 19F and 35B)
10F (1)	8841 (1)	none
20 (1)	8824 (1)	none
28 (1)	8823 (1)	none
38 (1)	393 (1)	393 (25A)
11A (1)	8836 (1)	none
11C (1)	8836 (1)	none
18B (1)	6239 (1)	ST6239 (18C)
18F (1)	8840 (1)	none
23B (1)	2911 (1)	2911 (23F)
33B (1)	4084 (1)	4084 (3, 10A, NT)
35F (1)	8832 (1)	none

1 Other serotypes associated with this sequence type (as listed in the MLST global database [28]) but not identified in this study, are within parantheses.

Sequence types representing isolates with reduced susceptibility to penicillin are in bold typeface.

### Antimicrobial susceptibility

For those PCV isolates with complete MLST profiles, 640 belong to clonal complexes/singletons representing at least one penicillin non-susceptible isolate (Table 1). Reduced susceptibility to penicillin was detected in 481 of those 640 isolates, of which 192 were multidrug resistant. Serotype 14, 19A and 19F had the largest proportion of isolates that were non-susceptible to penicillin (95% [109/115], 91% [119/131] and 89% [41/46], respectively) (Table 1 and 3). Non-susceptible isolates were common among serotype 14 and 19F isolates belonging to multiple clonal complexes. Except for one serotype 6B isolate, all other isolates belonging to CC230 (n = 49 for serotype 14 and 1 each for 19F and 23F) were multidrug resistant. All isolates belonging CC320 were also multidrug resistant (n = 23 and 1 isolate for serotype 19F and 23F, respectively).

**Table 3:** Serotype and clonal complex distribution of penicillin non-susceptible (PNS) and multidrug-resistant (MDR) isolates causing invasive pneumococcal disease in South Africa, 2007.

Serotype	Clonalcomplex^1^	No. ofisolates	No. of PNSisolates	No. of MDRisolates
			(%)^2^	(%)^3^
4	32	32	32 (100)	2 (6)
	63	1	1 (100)	1 (100)
6A	473	37	2 (5)	0
	1094	19	14 (74)	2 (11)
	156	23	8 (35)	1 (4)
	7084	2	1	0
	Singletons	8	3	0
6B	2421	26	19 (73)	4 (15)
	156	27	20 (74)	4 (15)
	473	12	9 (75)	2 (17)
	1094	8	6 (75)	0
	230	4	3 (75)	0
	242	7	6 (86)	4 (57)
	6236	2	2 (100)	0
	1381	5	3 (60)	0
	6393	2	2 (100)	0
	2185	2	1 (50)	0
	193	1	1 (100)	0
	439	1	1 (100)	0
	1016	1	1 (100)	0
	3594	1	1 (100)	0
	Singletons	15	10	0
9V	4881	40	14 (35)	1 (3)
	156	19	13 (68)	0
	473	1	1 (100)	1 (100)
	706	1	1 (100)	0
14	230	49	49 (100)	49 (100)
	63	25	22 (88)	20 (80)
	15	30	29 (97)	29 (97)
	156	2	1 (100)	1 (100)
	242	1	1 (100)	1 (100)
	2421	1	1 (100)	1
	6393	1	1 (100)	1
	Singletons	6	5	4
19A	2062	109	100 (92)	7 (6)
	156	9	9 (100)	1 (11)
	1381	1	1 (100)	0
	2416	1	1 (100)	0
	2421	1	1 (100)	0
	Singletons	10	7	1
19F	320	23	23 (100)	23 (100)
	347	6	6 (100)	1 (17)
	420	5	4 (80)	0
	2067	2	1 (50)	0
	81	1	1 (100)	0
	230	1	1 (100)	1 (100)
	Singletons	6	5	3
23F	242	25	16 (64)	16 (64)
	156	12	8 (67)	1 (8)
	81	4	4 (100)	4 (100)
	230	1	1 (100)	1 (100)
	63	1	1	0
	320	1	1 (100)	1 (100)
	473	1	1 (100)	1 (100)
	Singletons	8	5	3
Total		640	481	192

1 Only clonal complexes/singletons with at least one penicillin non-susceptible (PNS) isolate are reflected in the table;

2 Percentage of isolates that are penicillin non-susceptible within the specified clonal complex,

3 Percentage of isolates that are multi-drug resistant (MDR) within the specified clonal complex.

Of the 134 non-PCV serotype isolates recovered from children <2 years, 16% (21/134) were non-susceptible to penicillin and, of these, 19% (4/21) were multidrug resistant. The majority (14/21, 67%) of penicillin non-susceptible non-PCV isolates belonged to serotype 15B and were also non-susceptible to trimethoprim/sulphamethoxazole (13/14, 93%) but none were multidrug resistant. Of the 74 isolates with MLST results, 9 were non-susceptible to penicillin, 8 of which belonged to serotype 15B (Table 2). A non-typeable isolate represented by ST344 was multidrug resistant.

## Discussion

Serotypes with low carriage detection rates such as serotype 1 are normally less diverse while the opposite is true for serotypes that are associated with carriage. Consistent with the literature, our serotype 1 and 5 isolates were the least diverse while the remaining PCV serotypes were more heterogeneous. Several globally disseminated clones described by the pneumococcal molecular epidemiology network (PMEN), were identified among our isolates [22]. Sweden^1^-27 (CC217) represented nearly all (98%) of our serotype 1 isolates. South Africa^6B^-8 (ST185), which was identified among our serotype 6B isolates recovered in the 1990’s, continues to circulate [23]. Denmark^14^-32 (CC230) was the most prevalent clone among serotype 14 isolates. ST271, which is a single-locus variant of the multidrug resistant Taiwan^19F^-14 (ST236), was prevalent among our serotype 19F isolates.

Some serotypes were represented mainly by clonal complexes that appear to be uncommon in other parts of the world. CC458 was the most common genotype among serotype 3 isolates, while Netherlands^3^-31 (CC180) only represented 15% of our isolates. Our earlier study on a random selection of South African serotype 3 isolates, collected from 2000 through 2005 [24] also showed the predominance of CC458 which accounted for 54% of serotype 3 isolates tested (84/155), while CC180 represented only 12% (18/155) of the isolates. CC458 has also been documented among serotype 3 isolates from Ghana, Egypt, Israel, Costa Rica and Lithuania [25]–[27]. CC5410, which represented the majority of our serotype 4 isolates, has only been reported in Mozambique [28]. Interestingly, the majority (83%) of our serotype 19A isolates were CC2062, which, to our knowledge, has only been reported from South Africa [28]. In our earlier study, CC2062 was a novel genotype identified among 20% (5/25) of multidrug resistant serotype 19A isolates that caused IPD from 1998 through 2004 [29]. Other than CC156, which represented only 7% (9/131) of our serotype 19A isolates, no other common serotype 19A genotype including CC199 (predominant in the US), CC230 (common in Spain and Kenya) and CC320 (common in Spain, Asia and France and the US), was identified among our serotype 19A isolates [30]–[33]. CC320, which was responsible for the post-conjugate vaccine increase in penicillin-resistant serotype 19A in the US [34], represented half of our serotype 19F isolates all of which were penicillin and multidrug resistant.

Although not always the case, isolates with the same sequence type (or clonal complex) are usually of the same serotype [35]. A new or unusual serotype-sequence type combination is therefore often an indication of capsular switching. Pneumococci are highly transformable and a single in vivo recombination event that resulted in both a change from PCV7 serotype 4 to non-PCV7 19A and penicillin non-susceptibility has previously been documented in the US [36]. We identified clonal complexes among our PCV serotypes that expressed more than one capsular type. Although some of these clonal complexes have previously been reported in more than one serotype, we identified new serotype-ST combinations. CC156 has been identified among isolates belonging to multiple serotypes including 6A, 6B, 6C, 19A, 19F and 23F [28]. Similarly, CC156 was represented among our isolates belonging to multiple serotypes but was more common among serotype 6A and 23F isolates. The majority of our serotype 7F isolates were CC218, which, based on data in the global MLST database at the time of writing, is mainly associated with serotype 12F [28]. Nevertheless, this clonal complex has previously been identified among 7F isolates from other parts of the world including Saudi Arabia, Germany and Chile [28]. CC230 is associated with multiple serotypes but has not been reported among serotype 6B isolates [28], however four of our serotype 6B isolates were CC230. Although ST63 is a globally disseminated serotype 15A clone, it has also been identified among other serotypes including serotype 14 [28]. It is therefore not surprising that more than 20% of our serotype 14 isolates were CC63. We also identified clonal complexes that are normally associated with PCV serotypes among some of our non-PCV isolates. A limitation of our study was that our data only includes isolates collected over one year; hence we were unable to determine how long these unusual serotype-clonal complex combinations have been circulating in South Africa.

Since genotypic changes may also occur temporally, either in the absence of any known pressure or due to other selective pressures such as antibiotic use, we were not able to determine such changes with only one year of MLST data. Changes associated with antibiotic pressure have been reported in South Korea and Israel before they routinely used PCV [11], [12]. In South Korea, an increase in pneumococcal disease caused by antibiotic resistant serotype 19A was associated with expansion of ST320. In Israel, penicillin and multidrug resistant pneumococci recovered from patients with acute otitis media increased significantly from less than 10% to greater than 50% and was associated with the introduction and proliferation of two clones (ST276 [CC230] and ST2928 [CC63]) not previously associated with drug resistance. Denmark and Scotland reported fluctuations in pneumococcal genotypes at times of limited antibiotic and PCV use [10], [37]. An increase in the proportion of serotype 19A disease was reported in Denmark while an increase in serotype 1, associated with ST306, was reported in Scotland [10], [37]. Our South African surveillance data for children <5 years also showed a significant increase in penicillin non-susceptible 19A from 18% in 2003 to 78% in 2008, prior to the introduction of PCV [17]. Although limited genotypic data are available for our serotype 19A isolates, our current and previous studies show that the majority of our 2007 penicillin non-susceptible serotype 19A isolates (100/119, 84%) were CC2062, which has circulated among our multidrug-resistant 19A isolates for at least a decade [29]. The increase in penicillin resistance from 2003 to 2008 is therefore likely due to acquisition of *pbp* genes from other penicillin non-susceptible strains and requires further investigation.

PMEN was initially established for surveillance of globally disseminated pneumococcal clones that are antibiotic resistant. The majority of South African non-susceptible isolates were related to PMEN clones. All serotype 14 and 23F isolates related to Denmark^14^-32 were multidrug resistant. All serotype 19F and 23F isolates belonging to CC320, which also represent Taiwan^19F^-14 (ST236), were also multidrug resistant. The majority (80%) of serotype 14 isolates related to Sweden^15A^-25 (CC63) were multidrug resistant. The majority (73%) of serotype 6B isolates related to South Africa^6B^-8 [ST185 (CC156)] were non-susceptible to penicillin.

The biggest limitation of PCV is its inability to protect against pneumococcal disease caused by non-vaccine serotypes and hence the possibility of replacement disease. In our study, serotype 15B was the most common serotype among non-PCV isolates from children <2 years and had the highest proportion of isolates that were non-susceptible to penicillin. Moreover, serotype 15B and 8 were among the top 20 serotypes that caused IPD among South African children in 2003 through 2008, prior to the introduction of PCV [17], and may therefore be poised to fill the niche vacated by PCV serotypes. Increases in the number of serogroup 15 cases have been reported in eight hospitals in the US following routine vaccination with PCV [38]. Although serotypes 7F and 22F were not common among disease-causing isolates in South Africa, they have previously been reported as replacement serotypes elsewhere and therefore need to be closely monitored [4], [5].

We were not able to compare sequence types between HIV and non-HIV patients as HIV status was only available for IPD cases reported from sentinel sites (approximately 30 of the 187 laboratories participating in the surveillance program). As a result, HIV status for the majority of cases was unknown and hence there were low numbers of cases with available viable isolates (n = 816 for HIV infected and n = 231 HIV uninfected cases and, of these, 269 and 85 had MLST data, respectively).

This study represents the baseline genetic structure within pneumococcal serotypes prior to routine PCV use in South Africa. The predominance of genotypes such as CC217 (serotype 1), CC458 (serotype 3), CC5410 (serotype 4) and CC2062 (serotype 19A) that appear to be uncommon in other parts of the world highlights the importance of molecular characterisation of pneumococci from South Africa. Moreover, major sequence types circulating among invasive and carriage isolates from other African countries are also different to major sequence types identified globally [39], [40]. These data highlight the importance of molecular data from the African continent. Comparing pre-PCV to post-PCV genotypes (work currently in progress) will potentially provide insight into the impact of PCV on our pneumococcal population.

## Supporting Information

Figure S1 **Population snapshot depicting clonal relationships between sequence types of serotype 1 pneumococci causing invasive disease in South Africa, 2007 (n = 146).**
(PDF)

Figure S2 **Population snapshot depicting clonal relationships between sequence types of serotype 3 pneumococci causing invasive disease in South Africa, 2007 (n = 93).**
(PDF)

Figure S3 **Population snapshot depicting clonal relationships between sequence types of serotype 4 pneumococci causing invasive disease in South Africa, 2007 (n = 81).**
(PDF)

Figure S4 **Population snapshot depicting clonal relationships between sequence types of serotype 5 pneumococci causing invasive disease in South Africa, 2007 (n = 18).**
(PDF)

Figure S5 **Population snapshot depicting clonal relationships between sequence types of serotype 6A pneumococci causing invasive disease in South Africa, 2007 (n = 95).**
(PDF)

Figure S6 **Population snapshot depicting clonal relationships between sequence types of serotype 6B pneumococci causing invasive disease in South Africa, 2007 (n = 113).**
(PDF)

Figure S7 **Population snapshot depicting clonal relationships between sequence types of serotype 6C pneumococci causing invasive disease in South Africa, 2007 (n = 11).**
(PDF)

Figure S8 **Population snapshot depicting clonal relationships between sequence types of serotype 7F pneumococci causing invasive disease in South Africa, 2007 (n = 29).**
(PDF)

Figure S9 **Population snapshot depicting clonal relationships between sequence types of serotype 9 V pneumococci causing invasive disease in South Africa, 2007 (n = 70).**
(PDF)

Figure S10 **Population snapshot depicting clonal relationships between sequence types of serotype 14 pneumococci causing invasive disease in South Africa, 2007 (n = 115).**
(PDF)

Figure S11 **Population snapshot depicting clonal relationships between sequence types of serotype 18C pneumococci causing invasive disease in South Africa, 2007 (n = 57).**
(PDF)

Figure S12 **Population snapshot depicting clonal relationships between sequence types of serotype 19A pneumococci causing invasive disease in South Africa, 2007 (n = 131).**
(PDF)

Figure S13 **Population snapshot depicting clonal relationships between sequence types of serotype 19F pneumococci causing invasive disease in South Africa, 2007 (n = 46).**
(PDF)

Figure S14 **Population snapshot depicting clonal relationships between sequence types of serotype 23F pneumococci causing invasive in South Africa, 2007 (n = 59).**
(PDF)

Table S1 **Pneumococcal conjugate vaccine serotype isolate selection for multilocus sequence typing, by patient age category and serotype, South Africa, 2007.**
(PDF)
