# Supplementary figures and images for: Population Snapshot of Streptococcus pneumoniae Causing Invasive Disease in South Africa Prior to Introduction of Pneumococcal Conjugate Vaccines
Source: PLoS One. 2014 Sep 18;9(9):e107666. doi: 10.1371/journal.pone.0107666 (PMC4169438; doi:10.1371/journal.pone.0107666)

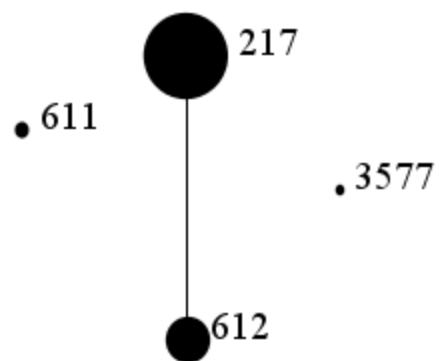

Figure S1

Supplement: Figure S1 — Population snapshot depicting clonal relationships between sequence types of serotype 1 pneumococci causing invasive disease in South Africa, 2007 (n = 146). (PDF) [file pone.0107666.s001.pdf]

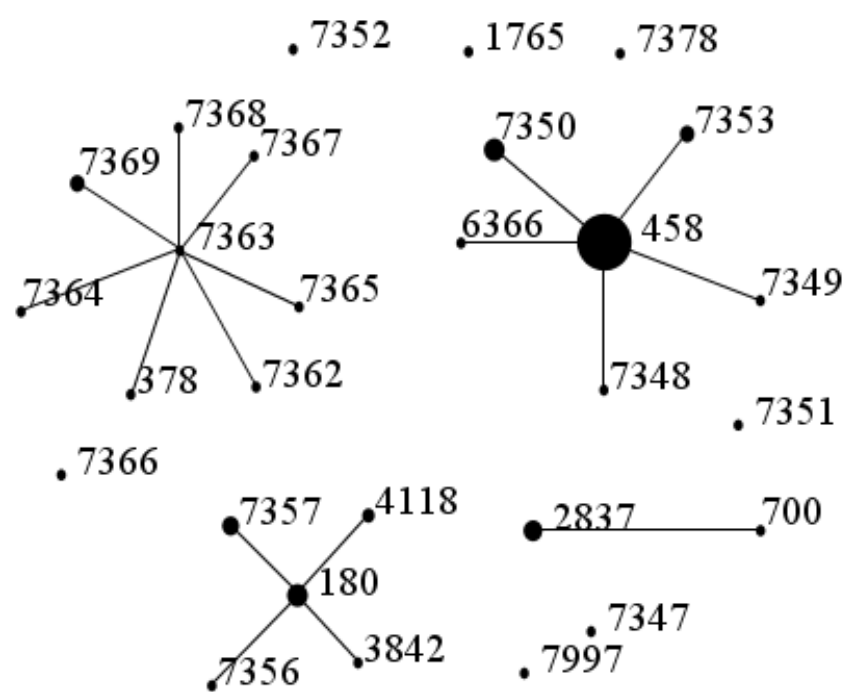

Figure S2

Supplement: Figure S2 — Population snapshot depicting clonal relationships between sequence types of serotype 3 pneumococci causing invasive disease in South Africa, 2007 (n = 93). (PDF) [file pone.0107666.s002.pdf]

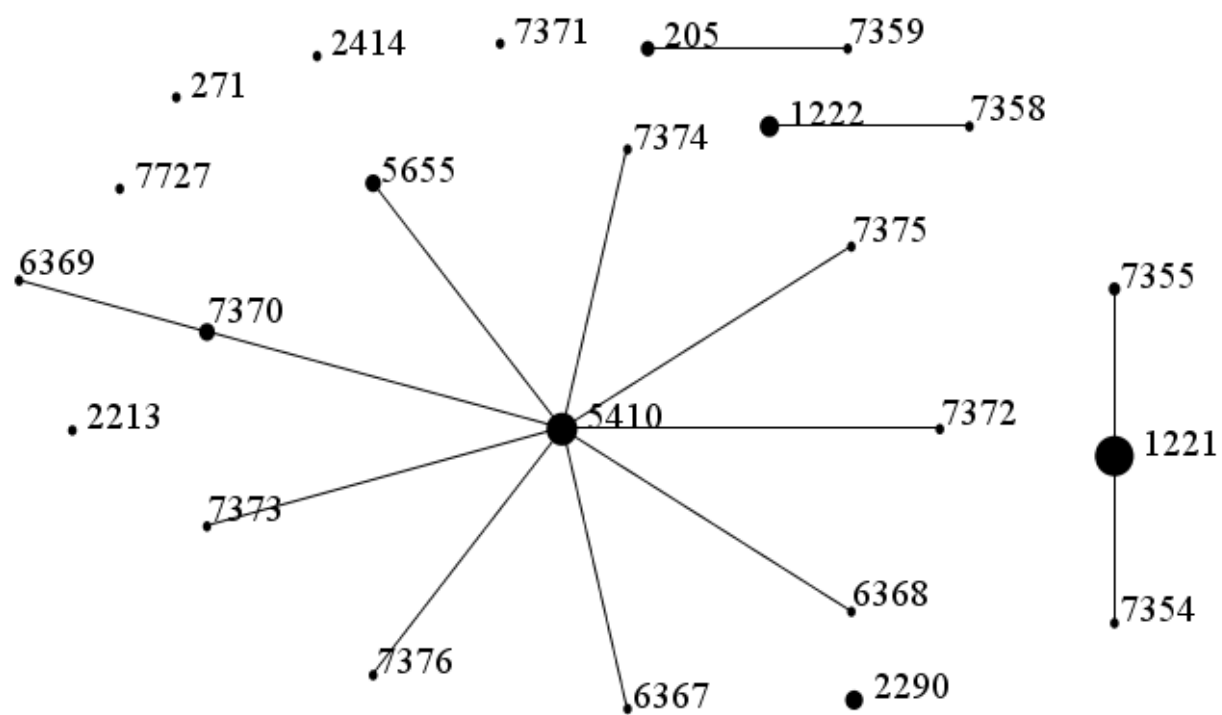

Figure S3

Supplement: Figure S3 — Population snapshot depicting clonal relationships between sequence types of serotype 4 pneumococci causing invasive disease in South Africa, 2007 (n = 81). (PDF) [file pone.0107666.s003.pdf]

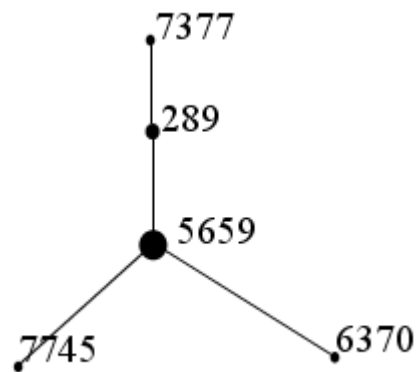

Figure S4

Supplement: Figure S4 — Population snapshot depicting clonal relationships between sequence types of serotype 5 pneumococci causing invasive disease in South Africa, 2007 (n = 18). (PDF) [file pone.0107666.s004.pdf]

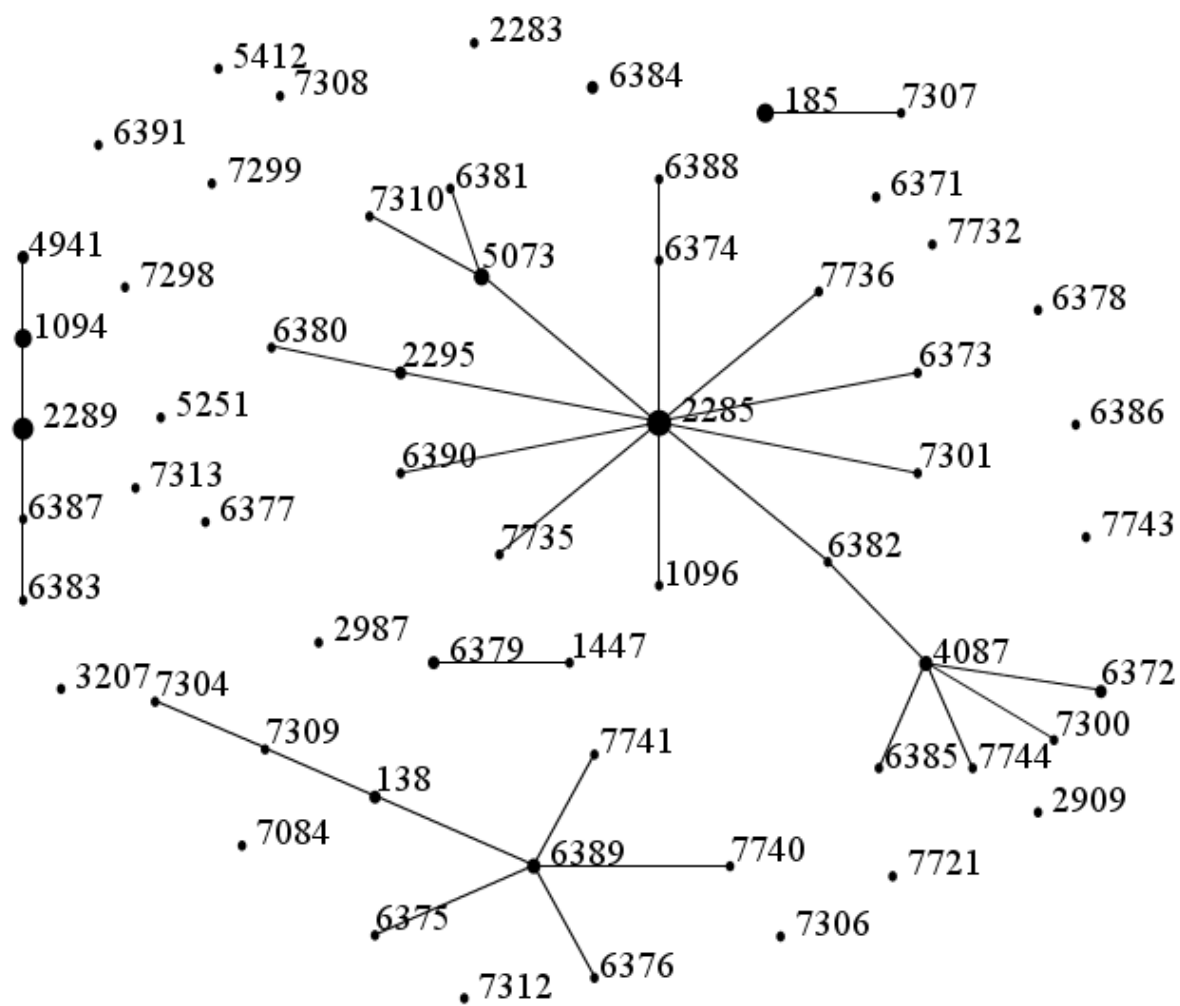

Figure S5

Supplement: Figure S5 — Population snapshot depicting clonal relationships between sequence types of serotype 6A pneumococci causing invasive disease in South Africa, 2007 (n = 95). (PDF) [file pone.0107666.s005.pdf]

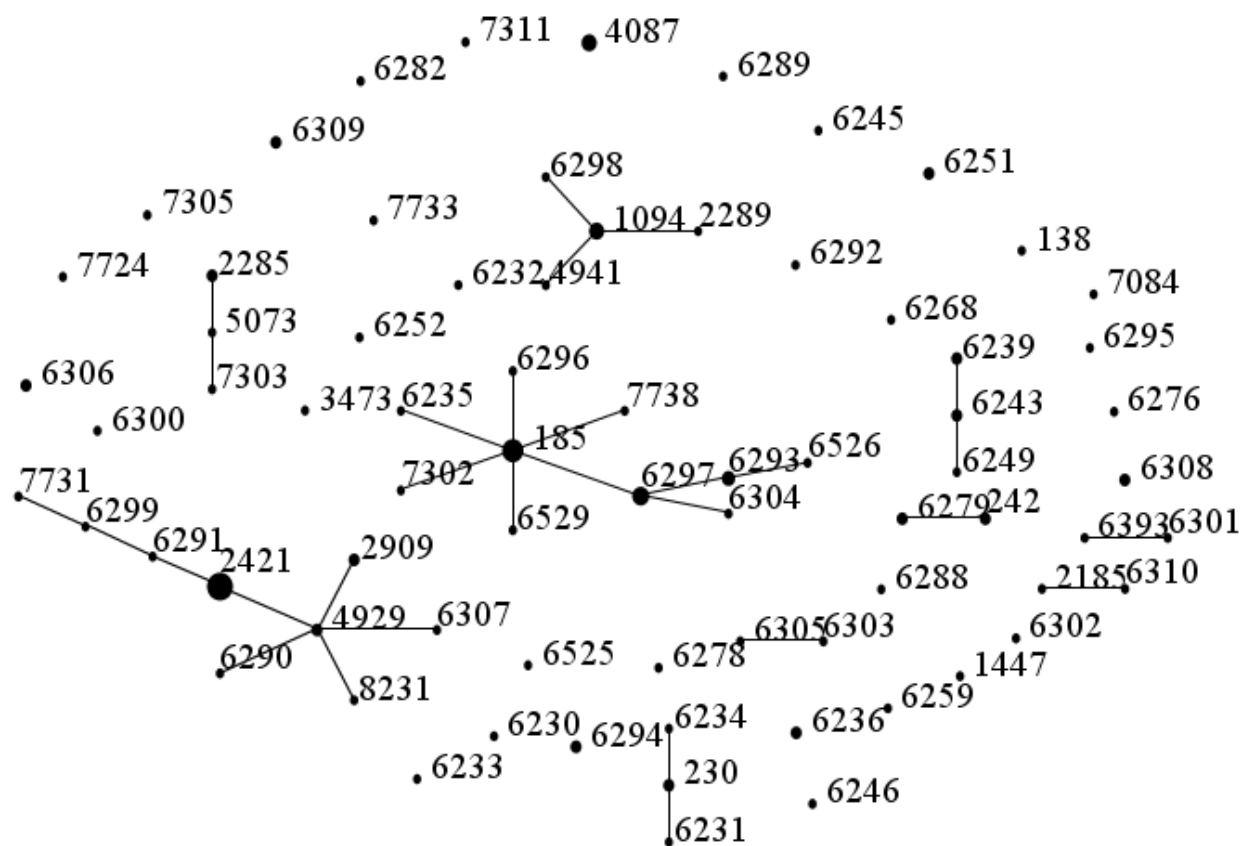

Figure S6

Supplement: Figure S6 — Population snapshot depicting clonal relationships between sequence types of serotype 6B pneumococci causing invasive disease in South Africa, 2007 (n = 113). (PDF) [file pone.0107666.s006.pdf]

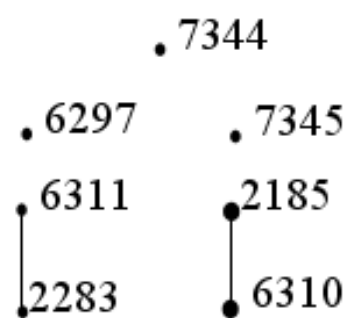

Figure S7

Supplement: Figure S7 — Population snapshot depicting clonal relationships between sequence types of serotype 6C pneumococci causing invasive disease in South Africa, 2007 (n = 11). (PDF) [file pone.0107666.s007.pdf]

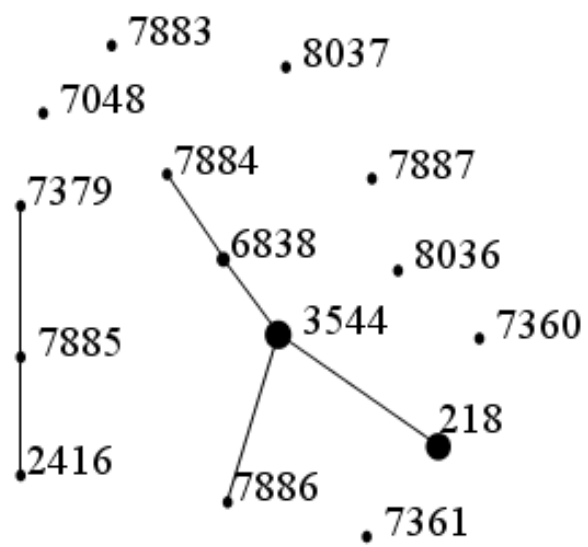

Figure S8

Supplement: Figure S8 — Population snapshot depicting clonal relationships between sequence types of serotype 7F pneumococci causing invasive disease in South Africa, 2007 (n = 29). (PDF) [file pone.0107666.s008.pdf]

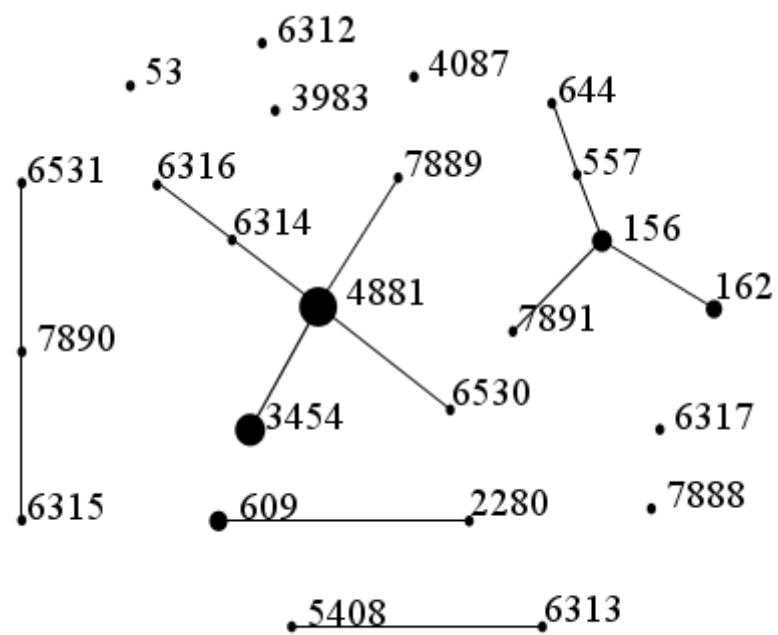

Figure S9

Supplement: Figure S9 — Population snapshot depicting clonal relationships between sequence types of serotype 9 V pneumococci causing invasive disease in South Africa, 2007 (n = 70). (PDF) [file pone.0107666.s009.pdf]

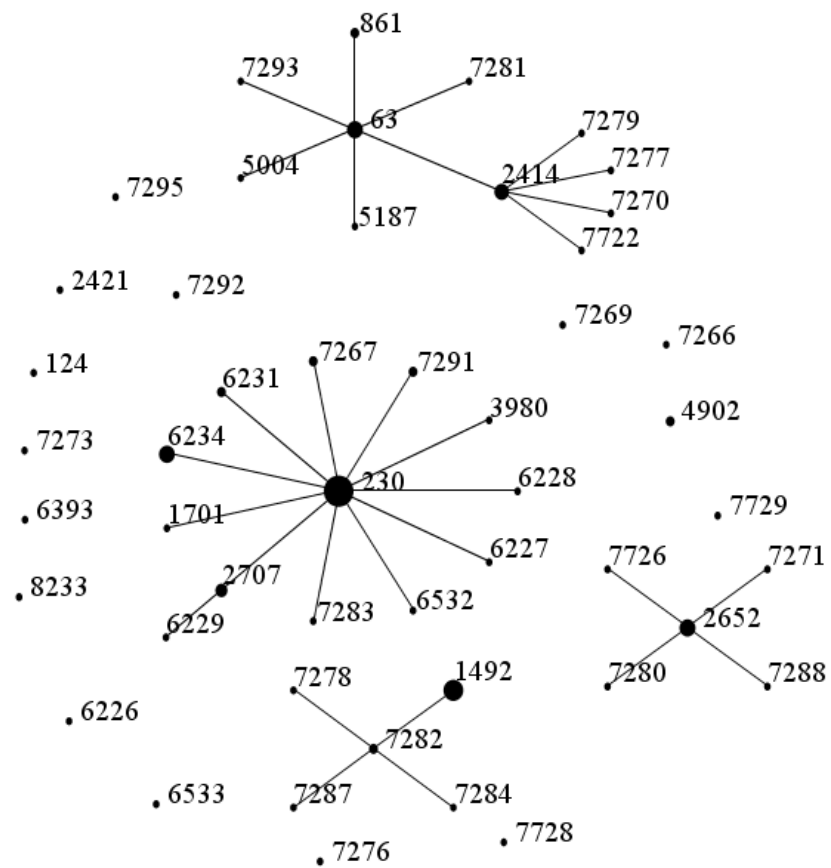

Figure S10

Supplement: Figure S10 — Population snapshot depicting clonal relationships between sequence types of serotype 14 pneumococci causing invasive disease in South Africa, 2007 (n = 115). (PDF) [file pone.0107666.s010.pdf]

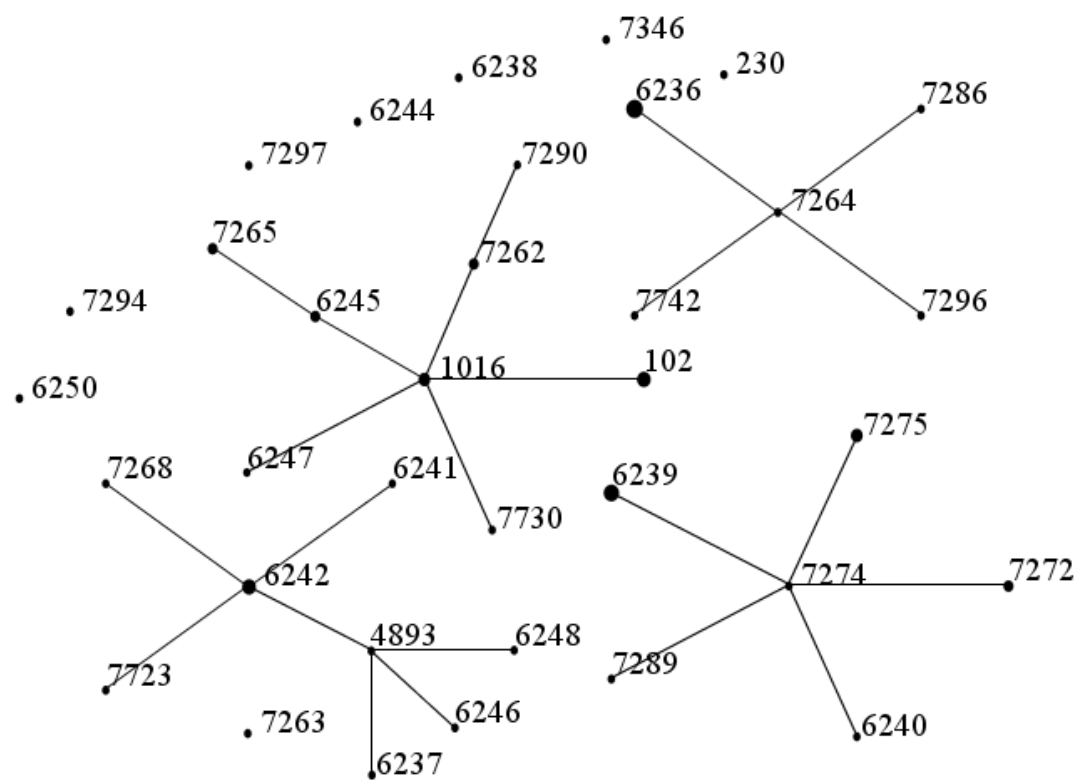

Figure S11

Supplement: Figure S11 — Population snapshot depicting clonal relationships between sequence types of serotype 18C pneumococci causing invasive disease in South Africa, 2007 (n = 57). (PDF) [file pone.0107666.s011.pdf]

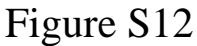

Supplement: Figure S12 — Population snapshot depicting clonal relationships between sequence types of serotype 19A pneumococci causing invasive disease in South Africa, 2007 (n = 131). (PDF) [file pone.0107666.s012.pdf]

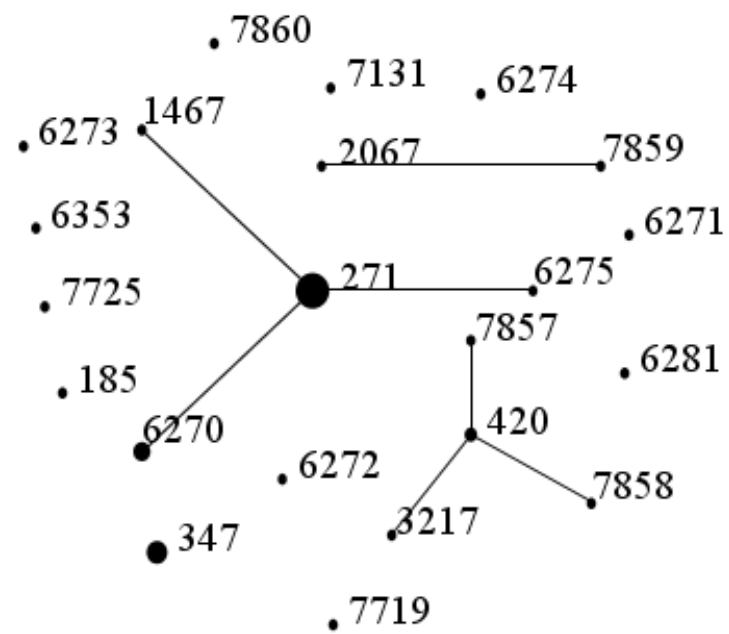

Figure S13

Supplement: Figure S13 — Population snapshot depicting clonal relationships between sequence types of serotype 19F pneumococci causing invasive disease in South Africa, 2007 (n = 46). (PDF) [file pone.0107666.s013.pdf]

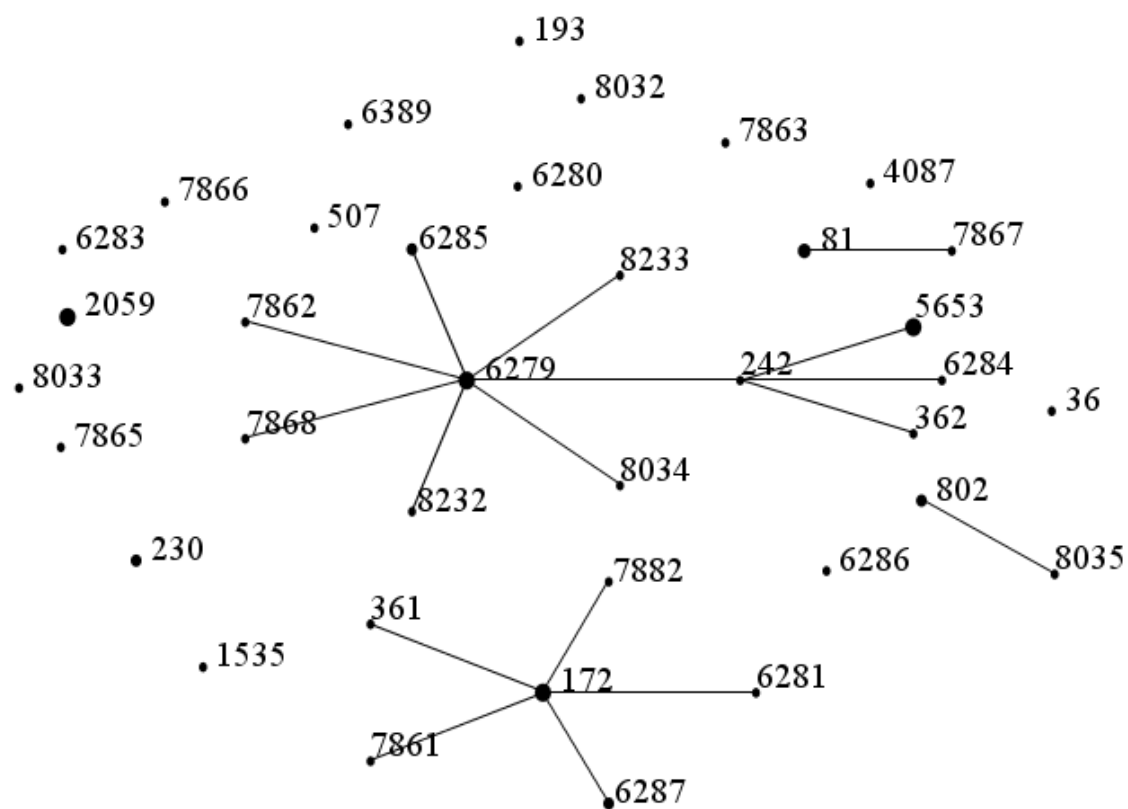

Figure S14

Supplement: Figure S14 — Population snapshot depicting clonal relationships between sequence types of serotype 23F pneumococci causing invasive in South Africa, 2007 (n = 59). (PDF) [file pone.0107666.s014.pdf]
