# Supplementary material for: Population Snapshot of Streptococcus pneumoniae Causing Invasive Disease in South Africa Prior to Introduction of Pneumococcal Conjugate Vaccines
Source: PLoS One. 2014 Sep 18;9(9):e107666. doi: 10.1371/journal.pone.0107666 (PMC4169438; doi:10.1371/journal.pone.0107666)
Supplement: Table S1 — Pneumococcal conjugate vaccine serotype isolate selection for multilocus sequence typing, by patient age category and serotype, South Africa, 2007. (PDF) [file pone.0107666.s015.pdf]

Table S1. Pneumococcal conjugate vaccine serotype isolate selection for multilocus sequence typing, by patient age category and serotype, South Africa, 2007

	<5 years			≥5 years		
Serotype	No. of isolates received	No. of isolates selected for MLST (%)[1]	No. of isolates with complete MLST profile (%)[2]	No. of isolates received	No. of isolates selected for MLST (%)[1]	No. of isolates with complete MLST profile (%)[2]
1	45	45 (100)	43 (96)	271	135 (50)	103 (76)
3	8	8 (100)	6 (75)	97	97 (100)	87 (90)
4	22	22 (100)	18 (82)	170	85 (50)	63 (74)
5	11	11 (100)	9 (82)	11	11 (100)	9 (82)
6A	136	68 (50)	48 (71)	133	66 (50)	47 (71)
6B	158	79 (50)	71 (90)	107	53 (50)	42 (79)
6C	5	5 (100)	4 (80)	10	10 (100)	7 (70)
7F	1	1 (100)	1 (100)	33	33 (100)	28 (85)
9V	31	31 (100)	24 (77)	64	64 (100)	46 (72)

14	158	79 (50)	63 (80)	137	67 (49)	52 (78)
18C	37	37 (100)	22 (59)	42	41 (98)	35 (85)
19A	84	84 (100)	68 (81)	192	96 (50)	63 (66)
19F	108	54 (50)	21 (39)	106	52 (49)	25 (48)
23F	123	61 (50)	18 (30)	137	66 (48)	41 (62)
Total	927	585 (63)	416 (71)	1510	876 (58)	648 (74)

[1]Percentage of isolates received that were selected for MLST [2]Percentage of isolates characterized that had a complete MLST profile
